# Effect of Crosslinking on the Properties and Stability of Andrographolide Solid Dispersions

**DOI:** 10.3390/pharmaceutics18101344

**Published:** 2026-10-21

**Authors:** Bingxin Liu, Xinli Liang, Guowei Zhao, Teng Ao, Qiang Zhang, Tangde Feng, Wei Dong

**Affiliations:** 1 Key Laboratory of Modern Preparation of Traditional Chinese Medicine, Ministry of Education, Jiangxi University of Chinese Medicine, Nanchang 330004, China; liubx9@163.com (B.L.); paln7@163.com (X.L.); 18279059079@163.com (T.A.); 13677027320@163.com (T.F.); sober96@foxmail.com (W.D.); 2 State Key Laboratory for the Modernization of Classical and Famous Prescriptions of Chinese Medicine, Nanchang 330004, China; 3 College of Chemistry and Life Sciences, Hubei University of Education, Wuhan 430205, China; qiang.zhang@hue.edu.cn

**Keywords:** crosslinking, crosslinking degree, solid dispersion, stability, effective dissolution, andrographolide

## Abstract

**Objectives**: Crosslinking enhances solid dispersion (SD) stability but often restricts drug release, and this trade-off remains unresolved. In this study, crosslinked polyvinyl alcohol–suberic acid ester (CPSE) carriers with different crosslinking degrees were prepared to investigate the effects of crosslinking on the properties, dissolution and stability of andrographolide (AG)-SDs, aiming to identify crosslinking conditions that ensure both effective dissolution and improved stability. **Methods**: CPSE was prepared at 110 °C or 130 °C for 4, 8 or 12 h with uncrosslinked polyvinyl alcohol (PVA) as the control. AG-CPSE-SDs and AG-PVA-SD were compared in terms of AG relative crystallinity (RC), surface free energy, hygroscopicity, dissolution and stability. Stability was evaluated after 30-day storage at 40 ± 2 °C/75 ± 5% RH. **Results**: Compared with AG-PVA-SD, AG-CPSE-SDs showed higher RC but lower surface free energy and hygroscopicity. SDs with lower crosslinking degrees had dissolution profiles similar to AG-PVA-SD, whereas those with higher crosslinking degrees showed significantly reduced dissolution rates. After storage, the dense network in highly crosslinked SDs hindered water entry and diffusion and restricted AG migration and recrystallization, thereby conferring better hygroscopic stability, interaction stability, recrystallization stability and dissolution stability. Notably, the SD using CPSE crosslinked at 130 °C for 4 h as the carrier balanced effective dissolution and enhanced stability. **Conclusions**: By tuning the crosslinking degree, SD stability can be improved without sacrificing drug dissolution. Moderate rather than maximal crosslinking achieves the optimal balance, providing a practical strategy for designing crosslinked carriers for SDs.

## 1. Introduction

Solid dispersion (SD) is an effective approach to enhance the solubility, dissolution rate and bioavailability of poorly soluble active pharmaceutical ingredient (API) [1,2,3]. SD offers numerous advantages: API is highly dispersed in the carrier material in an amorphous, molecular, or microcrystalline state, significantly increasing its dissolution rate and dissolution degree, enabling the formation of supersaturated solutions. The preparation process is straightforward and suitable to the production of oral solid dosage forms. In SD-related research and marketed products, polymers are the most used carrier materials [4,5,6,7] and play a critical role in determining SD stability. However, due to the strong hydrophilicity of polymers, SD is prone to moisture absorption during storage. Water acts as a plasticizer, leading to a decrease in the system’s *T*g, disruption of interactions between the polymer and the drug, reduced viscosity, and increased molecular migration rate. This results in aging phenomena such as increased hardness, crystal precipitation, or crystal coarsening, ultimately causing a reduction in SD solubility and dissolution rate, thereby diminishing the advantages of the solid dispersion method [8,9,10].

Compared with other solubilization methods, SD shows certain advantages in enhancing the AG solubility. Cyclodextrin inclusion is limited by the inclusion constant; superfine grinding leaves the drug in a crystalline state with limited dissolution improvement; nano-suspension and microemulsion require high surfactant usage and face difficulties in industrialization. SD can transform the drug into an amorphous or molecular state, significantly improving dissolution and bioavailability, with mature processes such as spray drying, showing greater industrialization potential. Its main limitation is the possible aging and recrystallization during storage.

Compared to conventional polymers, the structural characteristics of crosslinked polymers endow them with excellent performance in enhancing SD stability. Crosslinked polymers exhibit increased viscosity and reduced hygroscopicity. Their polymer chains interconnect and extend multidimensionally, forming a network-like spatial confinement structure that decreases the mobility of dispersed drug molecules, thereby delaying the recrystallization of SD and improving its stability [11,12,13]. However, the crosslinked structure also retards water penetration, leading to reduced system solubility. This may result in poor drug dissolution from the crosslinked polymer-SD system, which limits the application of crosslinked polymers in biopharmaceutical systems [14,15].

Andrographolide (AG) is a diterpenoid lactone compound that demonstrates special efficacy against various infectious diseases, including bacterial and viral upper respiratory tract infections, bacterial dysentery, viral pneumonia, and acute tonsillitis. Clinically, it is used as a preferred antiviral drug and is known as a “natural antibiotic” [16,17,18]. Currently, marketed oral formulations of AG include tablets, capsules, and drops. However, due to its high lipophilicity (logP = 2.63) and low water solubility (74 μg/mL), the bioavailability of AG after oral administration is low. Previous studies by our research group and related researchers have shown that formulating AG as SD can significantly improve its dissolution behavior [19,20,21]. However, the storage stability of AG-SD prepared with polymer carriers such as PEG and PVPK30 requires further improvement [22,23].

Therefore, in this study, crosslinked polyvinyl alcohol–suberic acid ester prepared under different crosslinking temperatures and durations was used as the carrier material, with polyvinyl alcohol serving as the reference carrier, to prepare AG-SD. We comparatively investigated the effects of crosslinked polymers and their crosslinking degrees on the physicochemical properties, dissolution behavior, and storage stability of AG-SD. The objective is to improve SD stability by using crosslinked polymers while ensuring the effective dissolution of drugs.

## 2. Materials and Methods

### 2.1. Materials

Andrographolide (AG, ≥98%, batch No. 5508-58-7) was purchased from Xi’an Haoxuan Biotechnology Co., Ltd. (Xi’an, China). Polyvinyl alcohol (PVA, batch No. P139537) was obtained from Shanghai Aladdin Biochemical Technology Co., Ltd. (Shanghai, China). Crosslinked polyvinyl alcohol–suberic acid ester (CPSE) was synthesized in the laboratory.

### 2.2. Preparation and Characterization of CPSE

Crosslinked polyvinyl alcohol–suberic acid ester (CPSE) was prepared via thermal crosslinking. PVA (2.00 g) and suberic acid (1.00 g) were accurately weighed at a mass ratio of 2:1 and transferred into a three-necked flask equipped with a condenser and magnetic stir bar. Subsequently, 50 mL of N,N-dimethylformamide (DMF) was added to the flask, and the mixture was stirred to achieve complete dissolution. The flask was placed in an oil bath maintained at 110 °C and 130 °C, respectively, for isothermal reactions of 4 h, 8 h and 12 h to obtain an original CPSE reaction solution. The CPSE solution was subjected to rotary evaporation at a water-bath temperature of 80 °C under reduced pressure to remove the solvent. The obtained crude product was mixed with 50 mL methanol under stirring to dissolve unreacted suberic acid, which was followed by suction filtration. This washing procedure was repeated three times. The collected solid was vacuum-dried in a DZF-6050 vacuum drying oven at 60 °C for 24 h. After grinding, CPSE powders were obtained, which were designated as CPSE1104, CPSE1108, CPSE11012, CPSE1304, CPSE1308 and CPSE13012, respectively.

Viscosity measurement: 0.2 g CPSE was dissolved in 5 mL DMF to prepare the CPSE solution. The viscosities of the CPSE solution and the above original CPSE reaction solution were measured using a Physica MCR rotational rheometer (Anton Paar GmbH, Graz, Austria). Instrument parameters: a sample volume of 300 μL was used with a CP50 rotor. The measurement was performed at 25 °C with a rotational speed of 100 r/min for 120 s, and data were collected from 20 points.

^1^H-NMR measurement: An appropriate amount of CPSE powder was dissolved in DMSO-d_6_ with TMS as the internal standard for the ^1^H-NMR characterization on an Avance III HD 600 MHz spectrometer (Bruker BioSpin GmbH, Fällanden, Switzerland). The test was carried out under isothermal conditions at 25 °C with a pulse delay of 1.00 s, spectral width of 12,019.23 Hz, 16 scans, and acquisition time of 1.08 s.

### 2.3. Preparation of AG-SDs

AG and the carrier material were accurately weighed at a 1:4 mass ratio and subsequently dissolved in 80% ethanol in an 80 °C water bath. The solution was spray-dried using a B-290 mini spray dryer (Büchi, Flawil, Switzerland) with an inlet temperature of 50 °C, feed rate of 1.7 mL/min, and nitrogen flow rate of 40 mL/min. The collected powder was stored in a vacuum desiccator, and the prepared SDs were denoted as AG-CPSE1104-SD, AG-CPSE1108-SD, AG-CPSE11012-SD, AG-CPSE1304-SD, AG-CPSE1308-SD, AG-CPSE13012-SD and AG-PVA-SD.

### 2.4. Preparation of PMs

AG and the carrier material were accurately weighed at a 1:4 mass ratio. They were then uniformly mixed using a vortex mixer, ground in a mortar, and subsequently sieved through a 60-mesh sieve to obtain an AG-carrier material–physical mixture (PM). The prepared PMs were designated as AG-CPSE1104-PM, AG-CPSE1108-PM, AG-CPSE11012-PM, AG-CPSE1304-PM, AG-CPSE1308-PM, AG-CPSE13012-PM, and AG-PVA-PM, respectively.

### 2.5. AG Content Determination

The content of AG was determined using a 1260 HPLC system (Agilent Technologies Inc., Santa Clara, CA, USA). The chromatographic conditions were as follows: Yilite C18 column (150 mm × 4.6 mm, 5 μm), mobile phase of methanol–water (60:40, *v*/*v*), flow rate of 1 mL/min, column temperature of 30 °C, UV detection at 225 nm, and injection volume of 10 μL. The method for AG determination was validated in preliminary experiments.

### 2.6. Fourier Transform Infrared Spectroscopy (FTIR) Analysis

FTIR spectra were recorded using a Spectrum Two FTIR spectrometer (PerkinElmer Inc., Waltham, MA, USA). Approximately 2–3 mg of the sample was mixed with dry potassium bromide and compressed at 10 tons for 2 min using an FW-4A powder compressing instrument (Uncommon Sci. Tech. Development Co., Ltd., Tianjin, China) to prepare discs. The spectra were scanned over a frequency range of 4000–400 cm^−1^ with a resolution of 4 cm^−1^, and 32 scans were accumulated for each disc.

### 2.7. X-Ray Diffraction (XRD) Analysis and Determination of Relative Crystallinity (RC)

XRD patterns were recorded on a D8 ADVANCE-D8X diffractometer (Bruker AXS, Karlsruhe, Germany) with Cu Kα radiation (λ = 1.54 Å; 40 kV × 40 mA); data were collected over 5–55° 2*θ* at a 0.02° step size and 8°/min scanning rate.

The *RC* of AG was determined by the internal standard method [22,24,25]: a fixed mass of sample and internal standard α-Al_2_O_3_ were homogenously mixed, and the XRD diffraction peak intensities of crystalline AG (*I*_AG_) and α-Al_2_O_3_ (*I*_iS_) in the mixture were calculated using Equation (1) and Equation (2), respectively.

*I*_AG_ = *K*_AG_[(*w*_1_*w*_AG_)/(*w*_1_ + *w*_2_)]/(*μ*/*ρ*) (1)

*I*_iS_ = *K*_iS_[*w*_2_/(*w*_1_ + *w*_2_)]/(*μ*/*ρ*) (2)

where *w*_1_, *w*_2_ and *w*_AG_ represent the mass fraction of the test sample, α-Al_2_O_3_, and crystalline AG, respectively. *μ*/*ρ* is the average mass absorption coefficient of the multiphase mixture, and *K*_AG_ and *K*_iS_ are the absorption constants of crystalline AG and α-Al_2_O_3_, respectively. Dividing Equation (1) by Equation (2) yields Equation (3):

*I*_AG_/*I*_is_ = *w*_AG_(*K*_AG_*w*_1_)/(*K*_iS_*w*_2_) (3)

If *w*_1_/*w*_2_ is a constant, Equation (3) indicates that when a fixed proportion of α-Al_2_O_3_ is added to the test sample, the mass fraction *w*_AG_ of crystalline AG in the test sample is linearly related to the diffraction peak intensity ratio (*I*_AG_/*I*_iS_).

AG-PM and α-Al_2_O_3_ (2:1 mass ratio) were mixed uniformly and designated as AG-PM+α-Al_2_O_3_. The XRD peak intensities of crystalline AG and α-Al_2_O_3_ in AG-PM+α-Al_2_O_3_ were *I*_AG-PM_ and *I*_iS-PM_, respectively. Similarly, AG-SD and α-Al_2_O_3_ (2:1 mass ratio) were mixed uniformly as AG-SD+α-Al_2_O_3_ with corresponding peak intensities *I*_AG-SD_ and *I*_iS-SD_. Assuming 100% crystallinity of AG in AG-PM+α-Al_2_O_3_, the *RC* of AG in AG-SD was calculated via Equation (4):

(*I*_AG-SD_/*I*_iS-SD_)/(*I*_AG-PM_/*I*_iS-PM_) = *RC*/100 (4)

### 2.8. Differential Scanning Calorimeter (DSC) Analysis

DSC thermograms were acquired on a DSC 8000 (PerkinElmer Inc., Waltham, MA, USA), which was calibrated with indium and zinc. Approximately 8 mg sample was accurately weighed into a hermetically sealed aluminum pan, and thermal analysis was conducted at 10 °C/min under N_2_ (20 mL/min) from 30 to 260 °C.

### 2.9. Wettability and Surface Free Energy Measurement

Approximately 0.10 g of the sample was compressed into tablets at 10 tons using an FW-4A press. Contact angles were measured on a Dataphysics OCA20 (Stuttgart, Germany) with H_2_O and CH_2_I_2_ as probe liquids with three parallel measurements per sample. The surface tension parameters are listed in Table 1.

A contact angle (*θ*) forms when a liquid droplet contacts a solid surface, which is related to solid surface free energy (*γ*_s_), liquid surface free energy (*γ*_l_), and solid–liquid interfacial free energy (*γ*_sl_) via the Young equation (Equation (5)). Furthermore, according to the Owens–Wendt–Kaelble theory, surface free energy can be decomposed into polar ($\gamma^{p}$) and non-polar components ($\gamma^{d}$), as shown in Equations (6) and (7).

*γ*_s_ = *γ*_l_cos*θ* + *γ*_sl_ (5)

(6) $$\gamma_{l}=\gamma_{l}^{p}+\gamma_{l}^{d}$$

(7) $$\gamma_{s}=\gamma_{s}^{p}+\gamma_{s}^{d}$$

According to Fowkes, the polar component reflects intermolecular dipole and hydrogen bonding interactions, while the non-polar component reflects intermolecular dispersion forces. Combining Equations (5)–(7), the solid–liquid interfacial free energy (*γ*_sl_) can be calculated using Equation (8).

According to Fowkes, the polar component reflects intermolecular dipole and hydrogen bonding interactions, while the non-polar component reflects intermolecular dispersion forces [26,27]. Combining Equations (5)–(7), the *γ*_sl_ can be calculated using Equation (8):

(8) $$\gamma_{\mathrm{sl}}=\gamma_{l}+\gamma_{s}-2\sqrt{\gamma_{l}^{p}/\gamma_{s}^{p}}-2\sqrt{\gamma_{l}^{d}/\gamma_{s}^{d}}$$

A further simplification of Equation (8) yields Equation (9):

(9) $$\gamma_{l}\left(1+\cos\theta \right)/2\sqrt{\gamma_{l}^{d}}=\sqrt{\gamma_{s}^{d}}+\sqrt{\gamma_{s}^{p}}\sqrt{\gamma_{l}^{p}/\gamma_{l}^{d}}$$

Therefore, probe liquids with different *γ*_l_, $\gamma_{l}^{p}$ and $\gamma_{l}^{d}$ were used to measure their contact angles on the solid surface. According to Equation (9), a plot of $\gamma_{l}\left(1+\cos\theta \right)/2\sqrt{\gamma_{l}^{d}}$ versus $\sqrt{\gamma_{l}^{p}/\gamma_{l}^{d}}$ was constructed to obtain a linear relationship. The slope and intercept of the line were used to calculate $\gamma_{s}^{p}$ and $\gamma_{s}^{d}$, respectively. The sum of $\gamma_{s}^{p}$ and $\gamma_{s}^{d}$ yields the *γ*_s_.

### 2.10. Hygroscopicity Measurement

The freshly prepared AG-SD powder was vacuum-dried at room temperature for 24 h in a DZF-6050 oven. Approximately 1 g of sample was evenly spread in a petri dish and placed in a stability chamber (25 °C, 75 ± 5% RH). The sample mass was recorded at 0, 2, 4, 6, 8, 12, 24, 48, 60, 72, 84, 96, 108, 120, 132 and 156 h. The experiment was conducted in triplicate with a moisture absorption curve plotted.

### 2.11. Water Content

Approximately 1.0 g of the sample was spread in an aluminum pan and heated at 105 °C until constant weight on a HE53/02 moisture analyzer (Mettler Toledo Technology China Co., Ltd., Shanghai, China). Water content was determined directly from mass loss.

### 2.12. Dissolution Testing

Dissolution testing was performed via the paddle method (Pharmacopoeia of China, 2025 Edition) using a ZRS-8G dissolution tester (Tianda-Tianfa Tech., Tianjin, China). Approximately 0.75 g of sample was accurately weighed into dissolution vessels with 900 mL double-distilled water (*n* = 6) at 100 rpm and 37 ± 0.5 °C. The process was monitored for 2 h, and 1.5 mL samples were collected at 5, 10, 15, 30, 45, 60, 90, and 120 min, which were immediately replaced with equal-volume fresh medium. Samples were filtered through a 0.22 μm membrane, and AG concentration was determined by the aforementioned HPLC conditions.

### 2.13. Stability Test

The freshly prepared AG-SD powder was vacuum-dried at room temperature for 24 h in a DZF-6050 vacuum drying oven. Approximately 2 g of sample was evenly spread in a petri dish (thickness ≤ 10 mm) and placed in a stability chamber (40 ± 2 °C, 75 ± 5%RH). On day 30, the water content, FTIR, XRD and dissolution behavior of AG-SDs were determined.

## 3. Results

### 3.1. Physicochemical Properties of CPSE

**Figure 1:**
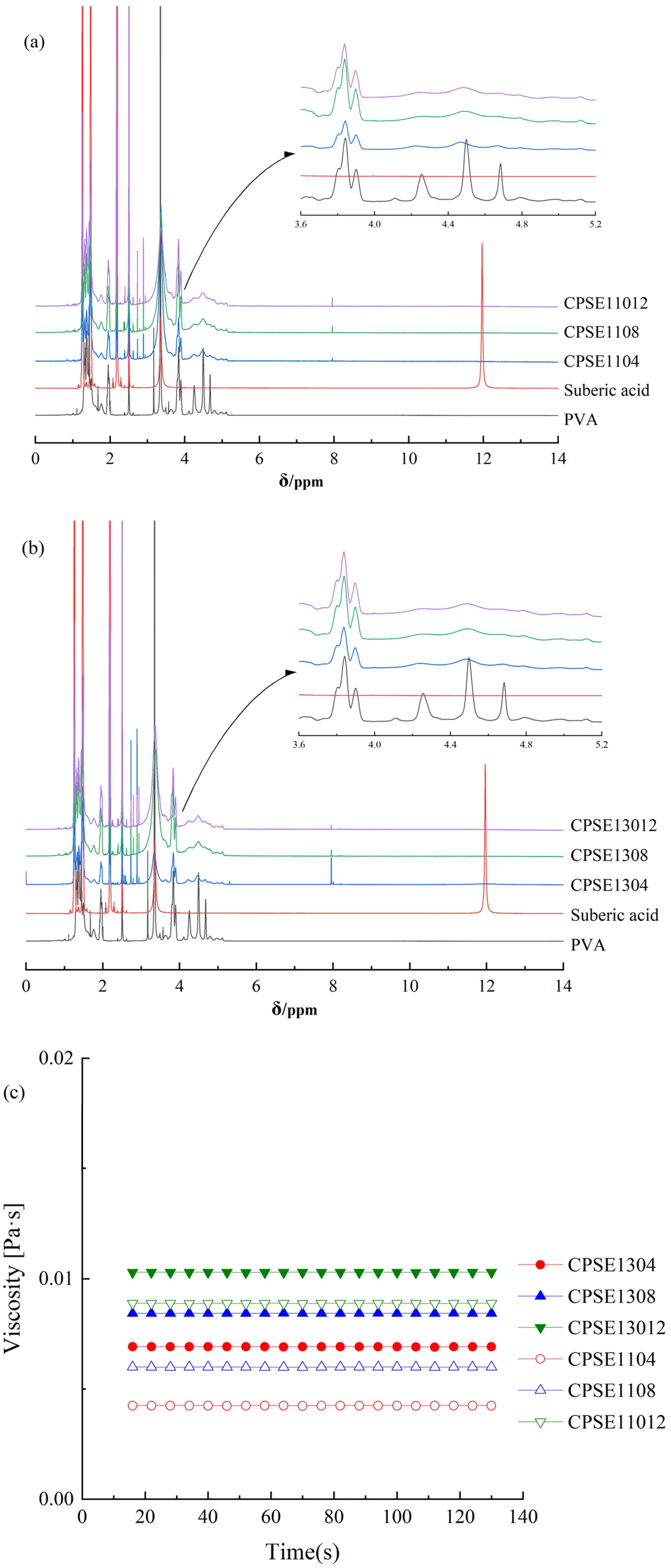

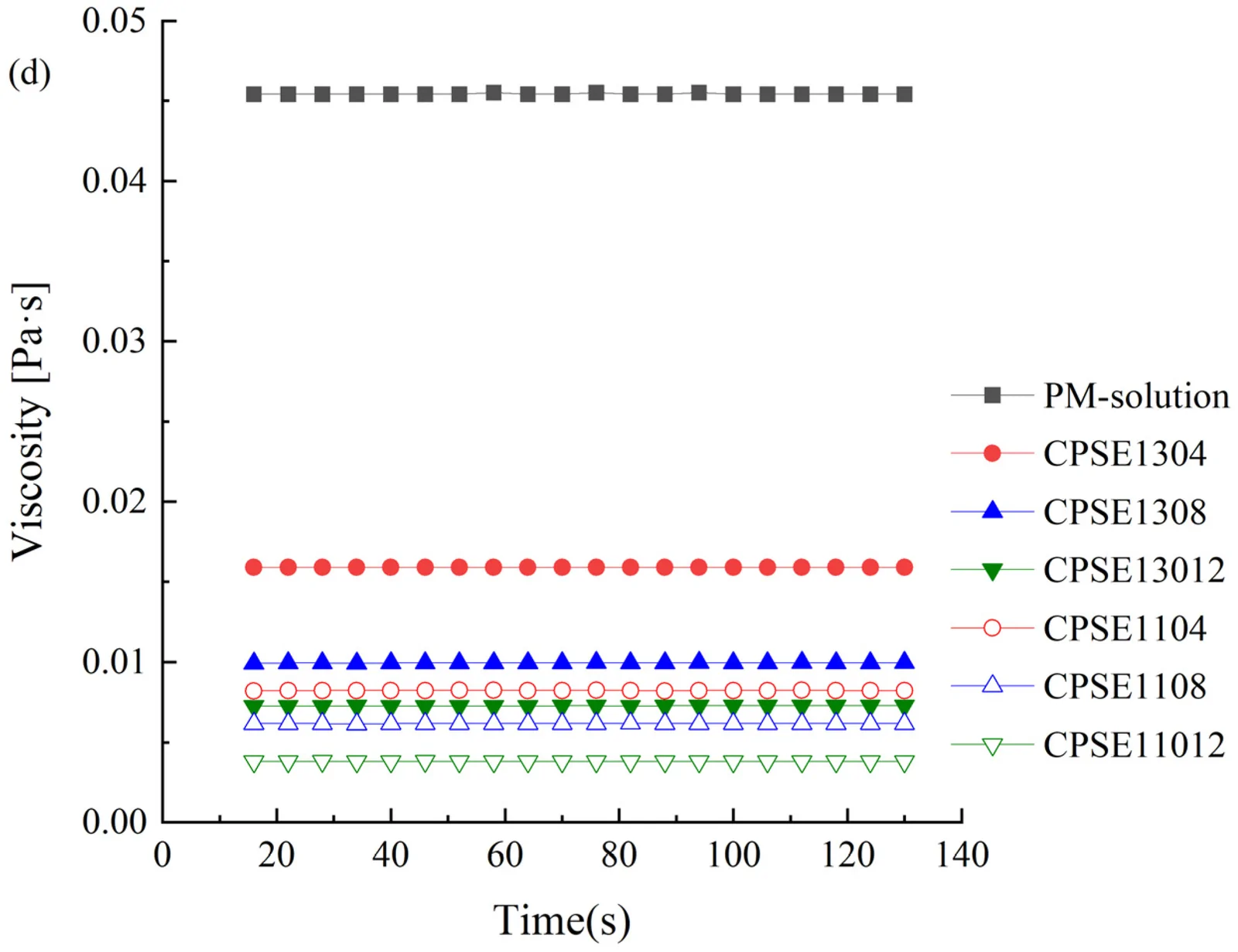
^1^H NMR spectra (**a**,**b**) of CPSE, viscosity curve of CPSE solution (**c**) and the original CPSE reaction solution (**d**).

Figure 1 shows the ^1^H NMR spectra and viscosity profiles of CPSE. As can be seen from Figure 1a,b, the characteristic peak assigned to the -CH-O-CO- proton of the esterified PVA backbone appeared at δ = 4.4–4.6 ppm, while the original -CH-OH signal of PVA at δ = 4.5 ppm disappeared, indicating the successful preparation of CPSE [28]. Moreover, the peak intensity corresponding to the ester bonds of CPSE and the viscosity curve (Figure 1c) of CPSE solution exhibited an increasing trend either by prolonging the crosslinking duration at a fixed crosslinking temperature or by raising the crosslinking temperature at a fixed crosslinking duration. This demonstrated that both extending crosslinking duration and/or elevating crosslinking temperature can enhance the crosslinking degree of CPSE. Nevertheless, although the crosslinking degree of CPSE rises with extended reaction time, the viscosity of the original CPSE reaction solution gradually decreases as crosslinking proceeds (Figure 1d). The esterification between PVA and suberic acid continuously produces water as a by-product, and all water generated during crosslinking remains within this homogeneous reaction system. As the reaction progresses, the accumulated by-product water progressively dilutes the CPSE system, which accounts for the continuous reduction in viscosity of the original CPSE reaction solution with increasing crosslinking time.

### 3.2. FTIR

As a non-invasive and highly sensitive analytical technique, infrared spectroscopy demonstrates unique advantages in elucidating intermolecular interactions and structural modifications. When compounds engage in intermolecular interactions such as hydrogen bonding and van der Waals forces, the vibrational frequencies of their functional groups undergo corresponding shifts, which are directly observable in their infrared spectra [29].

**Figure 2:**
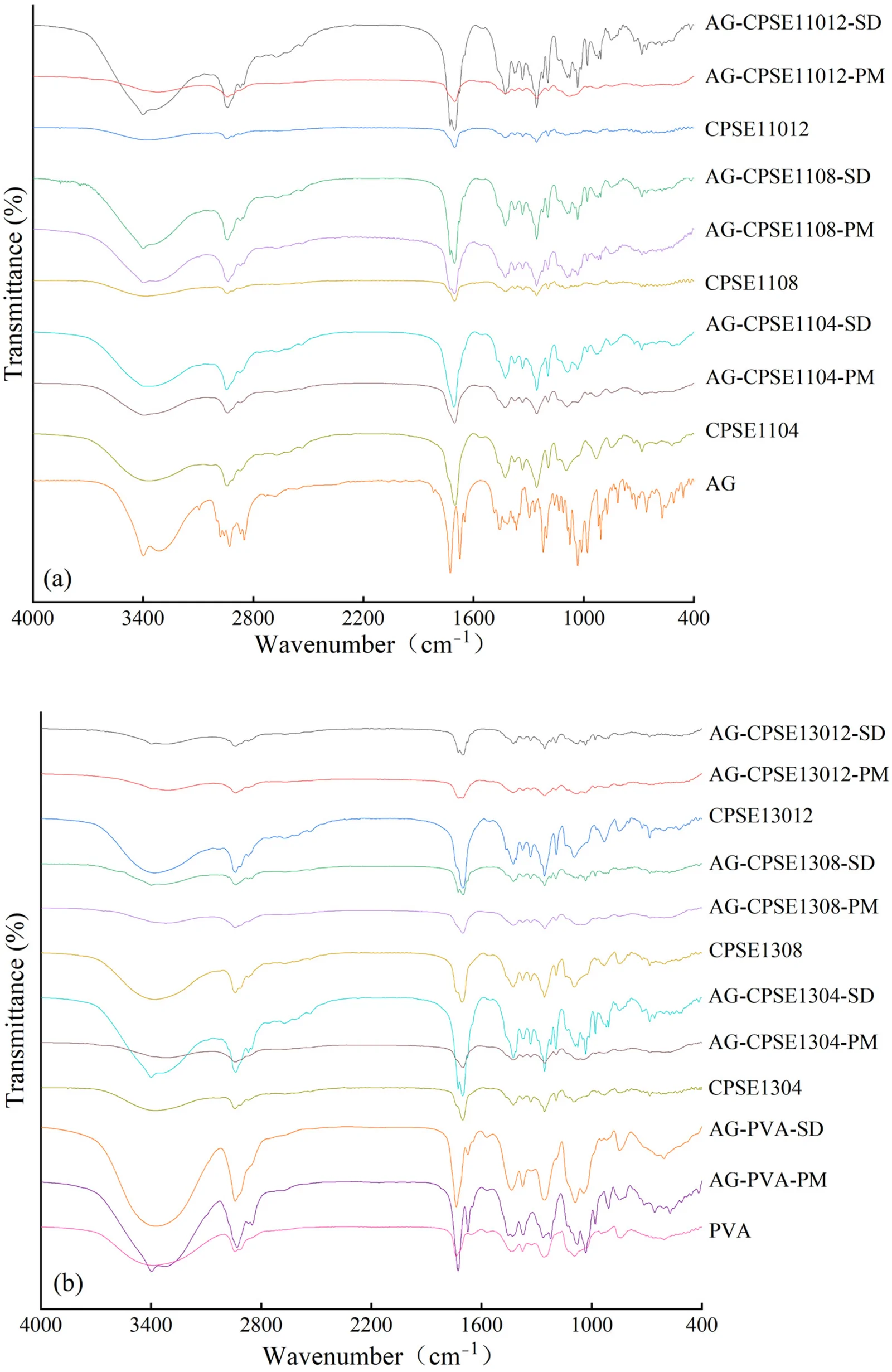
FTIR spectra of AG-SDs, AG-PMs, carrier materials and AG (**a**) CPSE110 as carrier material; (**b**) CPSE130 as carrier material.

Figure 2 shows the FTIR spectra of AG-SDs, AG-PMs, the carrier materials and AG. Among them, the broad band at 3200–3600 cm^−1^ was assigned to O–H stretching vibration; the band at 2850–2950 cm^−1^ was assigned to C–H stretching vibration; the band at 1700–1730 cm^−1^ was assigned to C=O stretching vibration; the band at 1600–1640 cm^−1^ was assigned to C=C stretching vibration; and the bands at 1000–1250 cm^−1^ were assigned to C–O–C and C–O stretching vibrations.

It can be seen from Table 2 that compared with AG, the -OH peak position in the AG-PMs was blue-shifted to different degrees, indicating that there was an intermolecular interaction between AG and the carrier material. Compared with the corresponding AG-PM, the -OH peak position in AG-SD exhibited a further blue shift, indicating that after being formulated into AG-SDs, the intermolecular interactions between AG and the carrier material were further enhanced due to the encapsulation effect. By comparing the -OH peak positions in AG-CPSE110-SD and AG-CPSE130-SD, it can be observed that compared to CPSE110, the increased crosslinking temperature and prolonged crosslinking duration in CPSE130 lead to a higher crosslinking degree, reduced -OH content, and consequently weakened interactions between AG and CPSE. This resulted in fewer degrees of blue shift in AG-CPSE130-SD.

In addition, compared with AG, the C=O stretching band at 1700–1730 cm^−1^ in AG-SDs was shifted and broadened, the C–O–C bands at 1000–1250 cm^−1^ also changed in intensity and profile, and the C=C band of AG at 1600–1640 cm^−1^ was markedly weakened, indicating that AG participated in intermolecular interactions, such as hydrogen bonding with the carrier.

### 3.3. XRD

**Figure 3:**
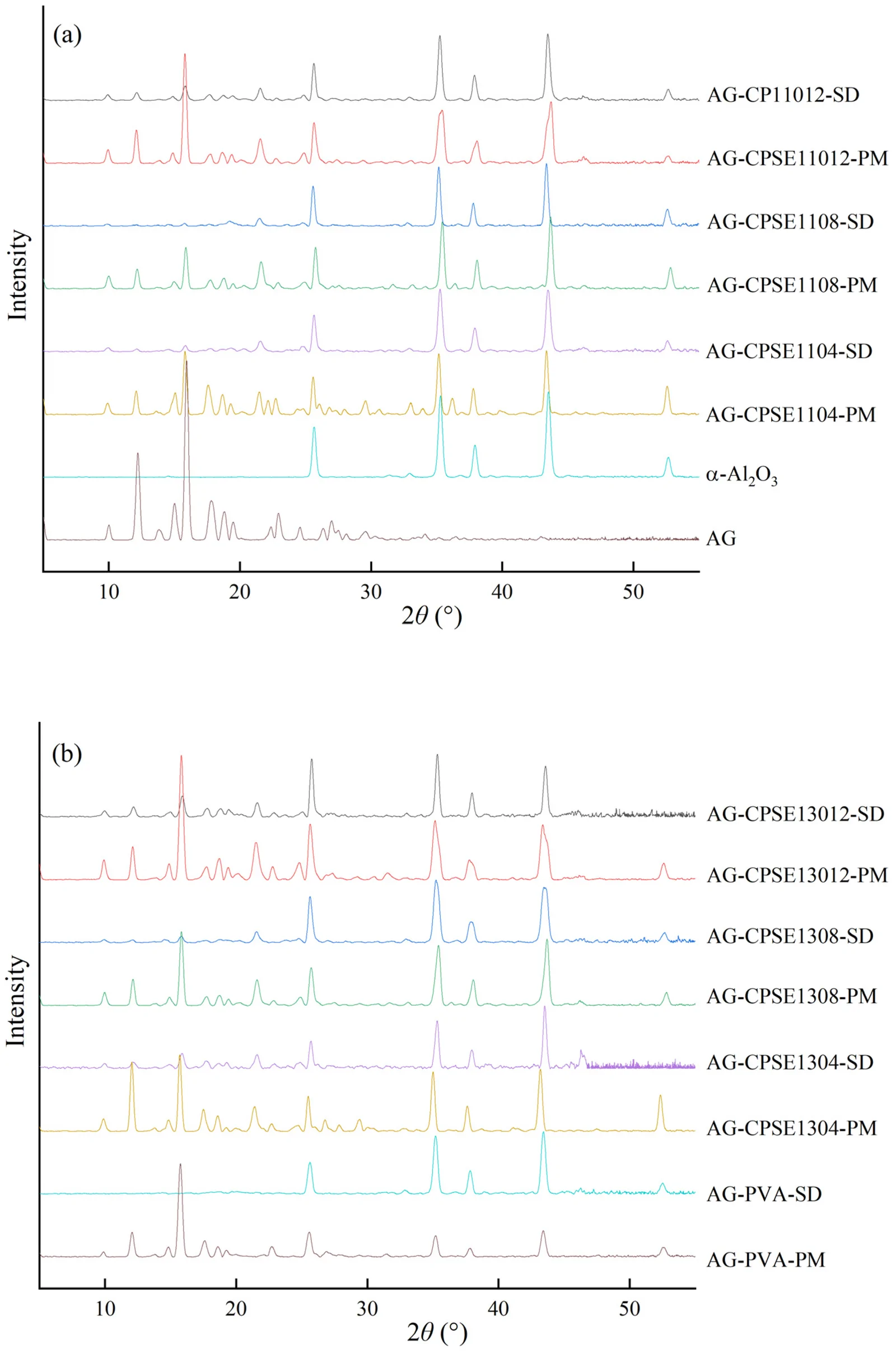
XRD diffractograms of AG-SDs, AG-PMs, carrier materials, pure AG and α-Al_2_O_3_. (**a**) CPSE110 as carrier material; (**b**) CPSE130 as carrier material.

XRD is the most convenient method for evaluating structure and crystallinity [25,30]. Figure 3 shows the XRD patterns of AG-SDs, AG-PMs, carrier materials, AG and α-Al_2_O_3_. The XRD pattern of pure AG exhibits multiple sharp diffraction peaks at 2*θ* of 9.78°, 12.12°, 14.91°, 15.77°, 17.73°, 18.61°, and 19.23°, corresponding to a typical crystalline structure. These diffraction peaks remain clearly visible in all AG-PMs. The AG diffraction peaks at 2*θ* of 12.2°, 15.0°, and 15.9° show no interference from the carrier material and internal standard substance (α-Al_2_O_3_). Therefore, their peak areas were selected to calculate the RC of AG in AG-SD, and the results are listed in Table 3.

As shown in Figure 3 and Table 3, the characteristic diffraction peaks of AG were not observed in AG-PVA-SD, indicating that AG existed in an amorphous state. In AG-CPSE-SDs, the characteristic diffraction peaks of AG were still visible, suggesting that AG existed in a partially crystalline and partially amorphous state with RC ranging from 10% to 30%.

A comparison between AG-CPSE110-SD and AG-CPSE130-SD revealed that the RC of AG-CPSE110-SDs ranged from 10% to 20%, while that of AG-CPSE130-SDs ranged from 20% to 30%. This difference can be explained from the perspective of the crosslinking degree of the carrier. During crosslinking, the crosslinker consumes the –OH groups on the carrier surface; therefore, the higher crosslinking temperature and longer crosslinking duration (CPSE130) lead to a higher crosslinking degree and a lower content of residual –OH groups on the carrier surface. Since these –OH groups are the primary sites for hydrogen bonding between AG and the carrier, their reduction weakens the AG–carrier intermolecular interactions, which is corroborated by the smaller blue shift of the –OH peak in the FTIR spectra. From a structural perspective, weaker AG–carrier interactions mean fewer “anchoring sites” that immobilize the AG molecules, thereby granting the AG molecules greater mobility during drying and solidification. This enhanced mobility allows the AG molecules to rearrange, promoting recrystallization and ultimately leading to a higher RC. Conversely, at a lower crosslinking degree (CPSE110), the abundant residual –OH groups form strong hydrogen bonds with AG, tethering the AG molecules in a disordered or semi-ordered state and suppressing their rearrangement, thus resulting in a lower RC. Therefore, the increase in RC from 10–20% (AG-CPSE110-SD) to 20–30% (AG-CPSE130-SD) is not merely a manifestation of weakened interactions but rather a structural consequence directly caused by the reduced hydrogen-bonding constraints that accompany a higher crosslinking degree. This trend is consistent with the FTIR analysis results.

### 3.4. DSC

**Figure 4:**
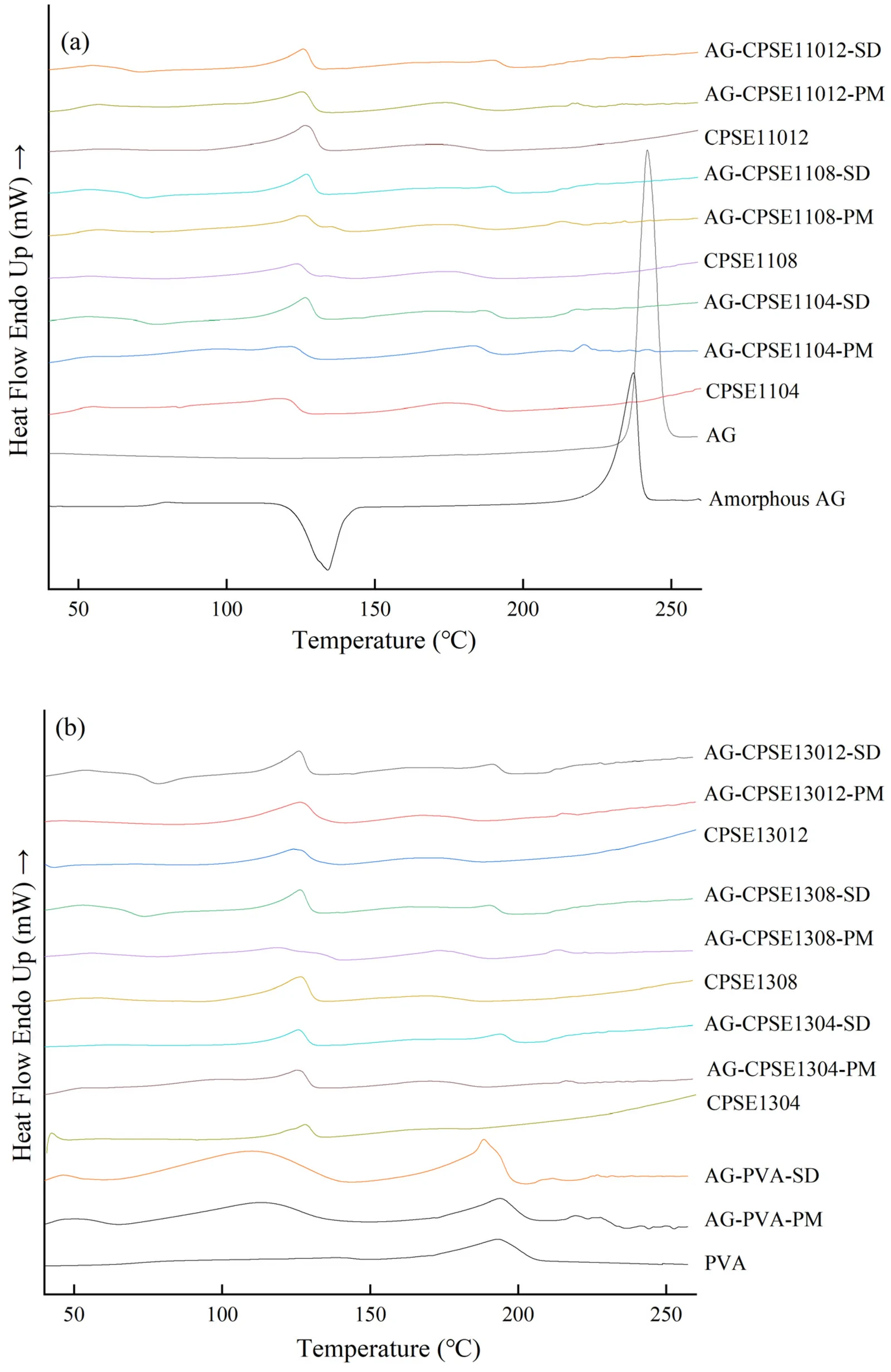
DSC thermograms of AG-SDs, AG-PMs, carrier materials, AG and amorphous AG. (**a**) CPSE110 as carrier material; (**b**) CPSE130 as carrier material. (↑ indicates an endothermic event).

Figure 4 shows the DSC thermograms of AG-SDs, AG-PMs, carrier materials, crystalline AG and amorphous AG. As shown in Figure 4, pure AG was a typical crystalline material with a sharp endothermic peak at its melting point of 242 °C. In AG-CPSE-PMs, a weak endothermic peak was observed between 210 and 220 °C, which was attributed to the influence of the carrier material, causing the endothermic peak of crystalline AG to shift to a lower temperature. Additionally, since the drug loading in AG-CPSE-PM was 20% and some crystalline AG may have dissolved in the molten carrier material, the endothermic peak of AG becomes broad and weak. Combined with the XRD results, the AG in AG-CPSE-SD existed in a partially amorphous state.

### 3.5. Wettability and Surface Free Energy

Table 4 presents the contact angle, $\gamma_{s}$, $\gamma_{s}^{p}$ and $\gamma_{s}^{d}$ for AG-SDs and AG-PMs. Compared to the corresponding AG-PM, AG-SD exhibited reduced water contact angles and increased polar components of surface free energy. This improvement was attributed to the encapsulation of the carrier material around the drug, which enhanced wettability and facilitates drug dissolution in AG-SD. However, the surface free energy of AG-SDs generally showed an increasing trend. Notably, the increase in surface free energy was most pronounced in AG-PVA-SD compared to AG-CPSE-SD (which used crosslinked polymer CPSE as the carrier), potentially exerting adverse effects on its stability.

As shown in Table 4, from CPSE1104/CPSE1304 to CPSE11012/CPSE13012, the water contact angles of AG-CPSE-PM and AG-CPSE-SD gradually increased, while the polar components of their surface free energy decreased accordingly. This phenomenon is attributed to the increased crosslinking degree resulting from higher crosslinking temperatures and longer crosslinking durations, which introduces more suberic acid (the crosslinking agent) into CPSE, thereby increasing the content of non-polar components in the polymer. Furthermore, the increase in crosslinking degree leads to a gradual reduction in surface free energy, which is conducive to maintaining the stability of AG-CPSE-SD. The observed trend of increasing contact angle and decreasing polar surface free energy with higher crosslinking degree is consistent with previous studies on crosslinked polymer systems. For instance, Kim et al. reported that the contact angle of crosslinked PVA membranes increases with the crosslinking degree [31], and Hyder et al. demonstrated that the surface energy decreases with increasing crosslinker concentration [32]. The polar component of surface free energy was found to decrease markedly upon crosslinking in PVA-SSA systems [33].

### 3.6. Hygroscopicity

**Figure 5:**
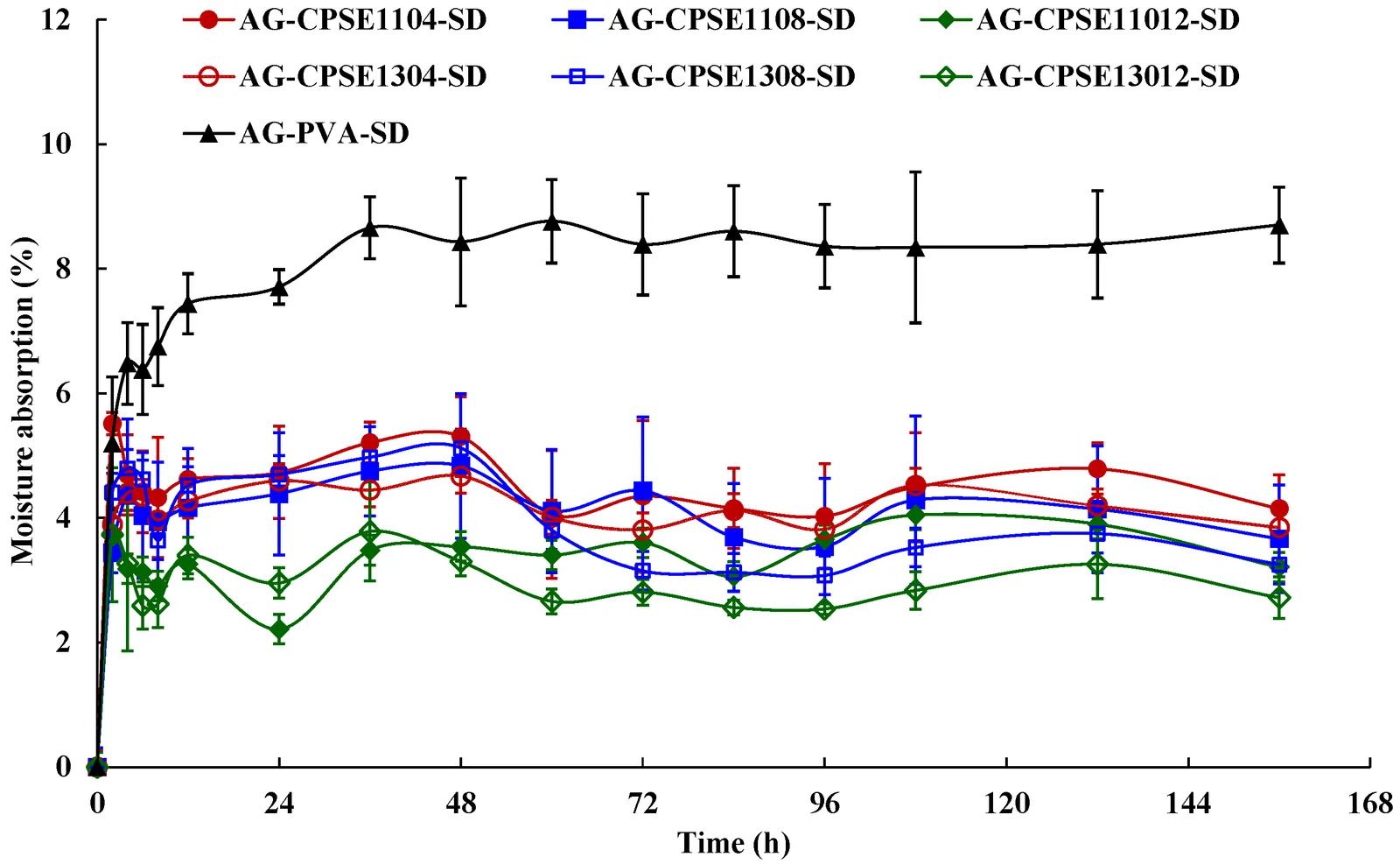
Moisture absorption curves of AG-SDs.

Hygroscopicity is a critical factor affecting the stability of SD. Figure 5 shows the moisture absorption curves of AG-SDs. As observed in Figure 5, the moisture absorption rate of AG-PVA-SD was significantly higher than that of AG-CPSE-SDs, indicating that the compact structure of the crosslinked polymer can markedly reduce the hygroscopicity of AG-CPSE-SDs prepared using this carrier material. To further investigate the effects of crosslinking temperature and duration on the hygroscopicity of AG-CPSE-SD, the difference factor method (*f*_1_) was employed to analyze the variability in moisture absorption curves [34]. The formula for *f*_1_ is shown in Equation (10):

(10) $$f_{1}=\frac{\sum_{t=1}^{n}\left|R_{t}-T_{t}\right|}{\sum_{t=1}^{n}R_{t}}\cdot 100$$

where *R*_t_ and *T*_t_ denote the moisture absorption rates of the reference sample and test sample at a given sampling time *t*, respectively. The parameter *n* represents the total number of sampling time points. A smaller *f*_1_ value indicates a higher similarity between the two moisture absorption curves. Specifically, *f*_1_ values ranging from 0 to 15 confirm the equivalence of the compared curves.

Table 5 lists the *f*_1_ values for the moisture absorption curves of AG-CPSE-SD. Comparing the effects of crosslinking duration, no significant difference was observed when prolonging the duration from 4 h to 8 h. However, prolonging the duration to 12 h resulted in a notable difference, indicating that only after sufficient crosslinking duration does the increased crosslinking degree significantly reduce the hygroscopicity of AG-CPSE-SD. For crosslinking temperature, significant differences in hygroscopicity attributable to temperature-induced variations in crosslinking degree were observed only when the crosslinking duration reached 12 h.

### 3.7. Dissolution

**Figure 6:**
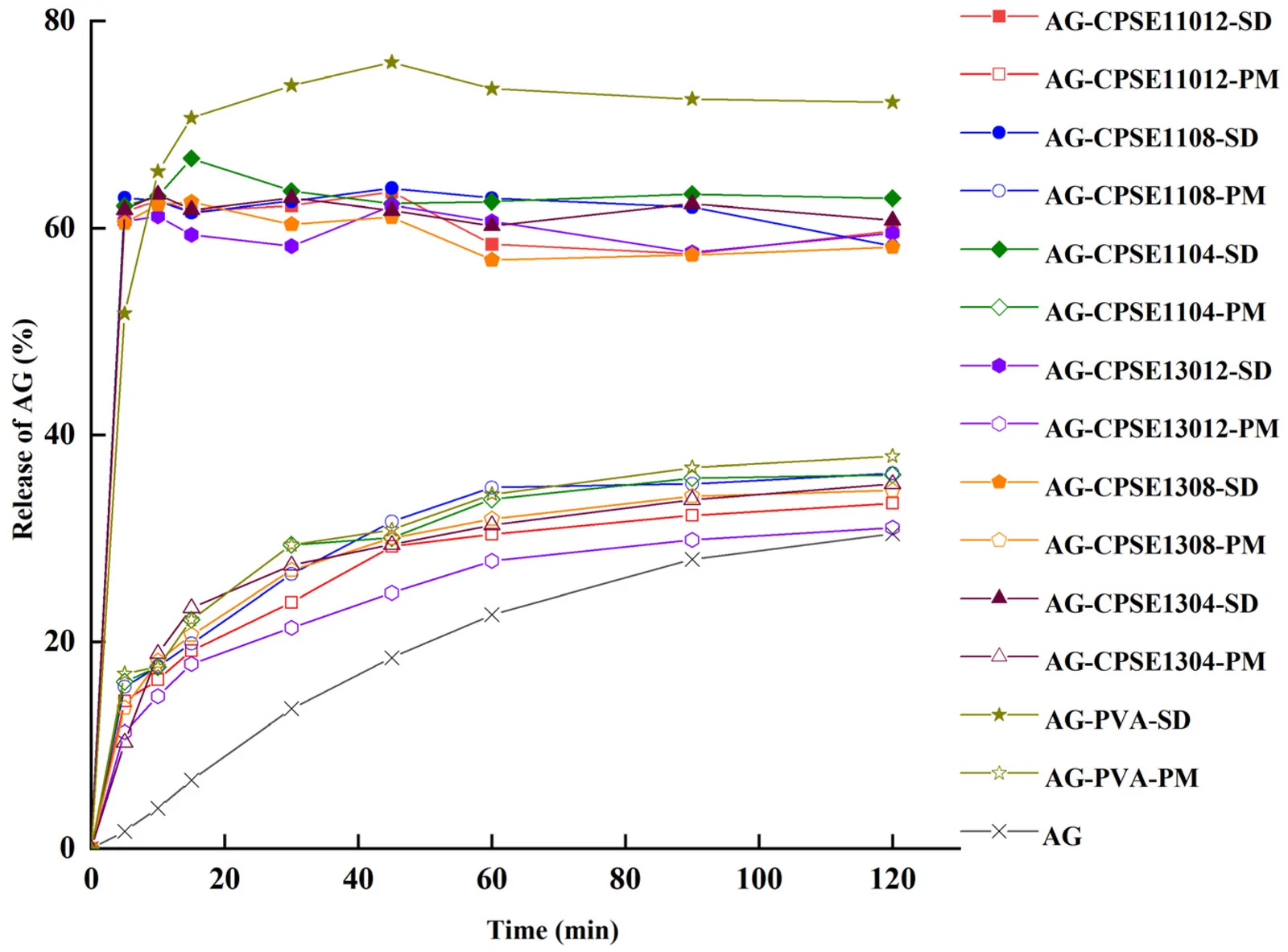
Dissolution profiles of AG-SDs, AG-PMs and pure AG.

The dissolution of oral solid dosage forms of poorly soluble drugs is a key step in the oral absorption process and an important parameter related to bioavailability [35,36]. Figure 6 shows the dissolution curves of AG-SDs, AG-PMs, and AG. As shown in Figure 6, the dissolution percentage of AG in water was only 30.46% at 120 min, while preparing AG into SDs significantly increased its dissolution percentage.

Compared to AG-PVA-SD, the dissolution curves of AG-CPSE-SDs, which used crosslinked polymer as the carrier material, showed a downward trend. It is worth noting that the dissolution curves of AG-CPSE11012-SD, AG-CPSE1308-SD and AG-CPSE13012-SD reached a maximum at approximately 45~60 min and then declined slightly. This behavior can be explained by the supersaturation phenomenon of SDs. In this study, the SDs produced supersaturation: pure AG dissolved only 30.46% at 120 min, whereas the SDs reached substantially higher dissolution percentages, indicating that the drug concentration in the medium exceeded its equilibrium solubility. However, a supersaturated solution is a metastable state that tends to revert to the crystalline state, and its maintenance depends strongly on the carrier. For these highly crosslinked carriers, XRD confirmed that a higher crosslinking degree leads to a higher relative crystallinity of AG, i.e., a larger fraction of AG exists in a partially crystalline state. Consequently, the dissolved AG may undergo recrystallization or precipitation once the solution approaches a supersaturated state. In addition, the denser crosslinked network restricts the diffusion of AG and may promote the re-adsorption of dissolved AG onto the carrier via hydrogen bonding with residual –OH groups. These effects together account for the slight decrease in AG concentration in the later stage of dissolution.

Further, the difference factor method outlined in Section 3.6 was used to study the effect of crosslinking on the dissolution behavior of AG-CPSE-SD, where *R*_t_ and *T*_t_ denote the dissolution percentage of the reference sample and test sample at a given sampling time *t*, respectively. The parameter *n* represents the total number of sampling time points, and the results are shown in Table 6. As seen in Table 6 (the prefix 30d of the names correspond to the stability study that will be commented later), taking AG-PVA-SD as the reference, the *f*_1_ values of AG-CPSE1104-SD, AG-CPSE1108-SD, and AG-CPSE1304-SD were all < 15, indicating that the dissolution curves of these three SDs were equivalent to that of AG-PVA-SD. In contrast, the *f*_1_ values of AG-CPSE11012-SD, AG-CPSE1308-SD, and AG-CPSE13012-SD were all > 15, indicating that the dissolution curves of these three SDs were not equivalent to that of AG-PVA-SD. This suggested that using crosslinked polymers with lower crosslinking temperatures and shorter crosslinking durations as carrier materials does not significantly affect the dissolution of AG-CPSE-SD.

### 3.8. Stability

#### 3.8.1. Hygroscopicity Stability

**Figure 7:**
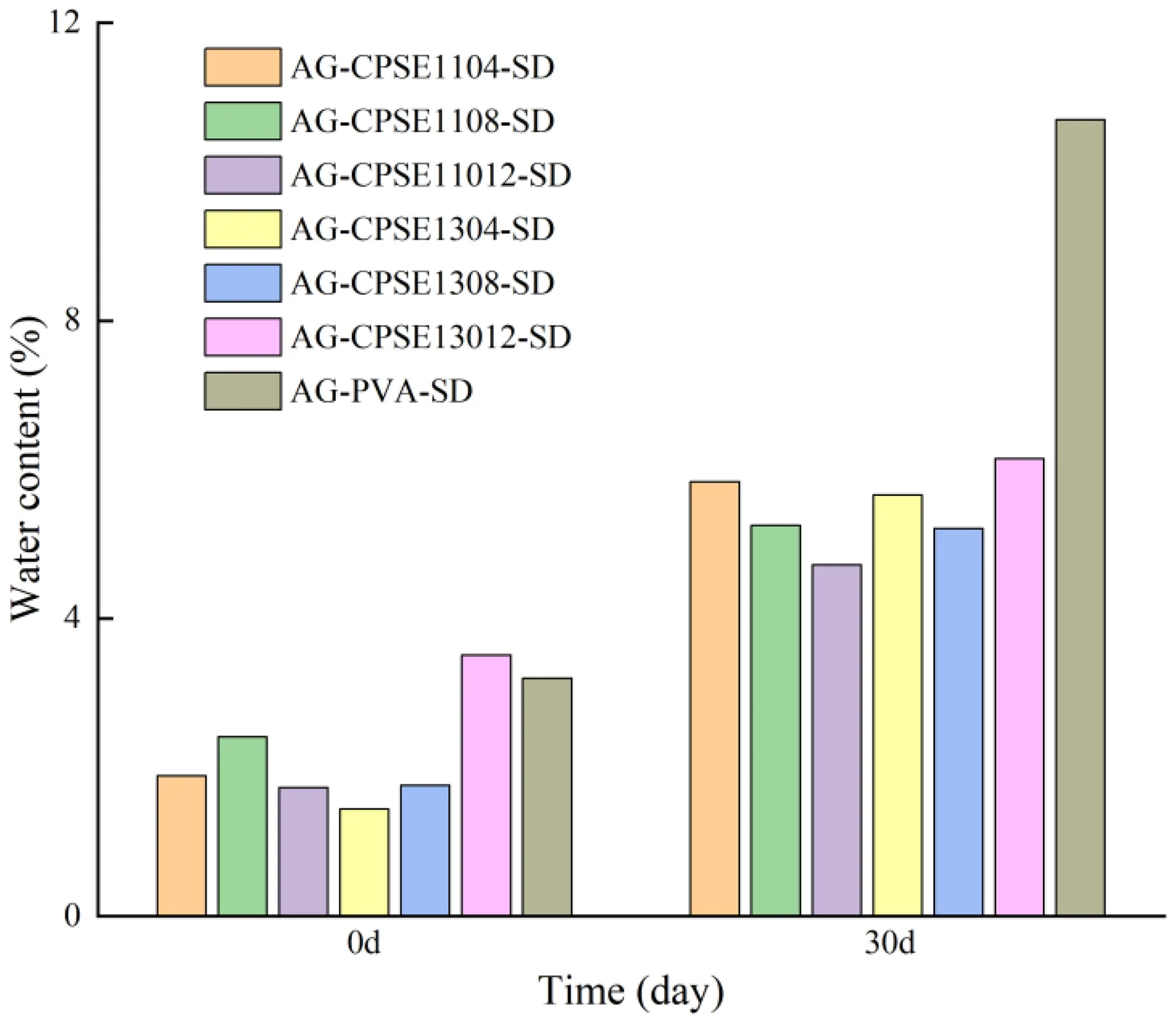
Moisture content of AG-SDs before and after 30 days of storage at 40 ± 2 °C/75 ± 5% RH.

Figure 7 shows the moisture content changes of AG-SD before and after 30 days of storage at 40 ± 2 °C/75 ± 5%RH. As shown in Figure 7, after 24 h of vacuum drying, the moisture content of freshly prepared AG-SD was less than 3%. After 30 days of storage under high temperature and humidity, the moisture content of all AG-SDs increased significantly. Among them, the moisture content of the six AG-CPSE-SDs was significantly lower than that of AG-PVA-SD.

#### 3.8.2. Intermolecular Interaction Stability

**Figure 8:**
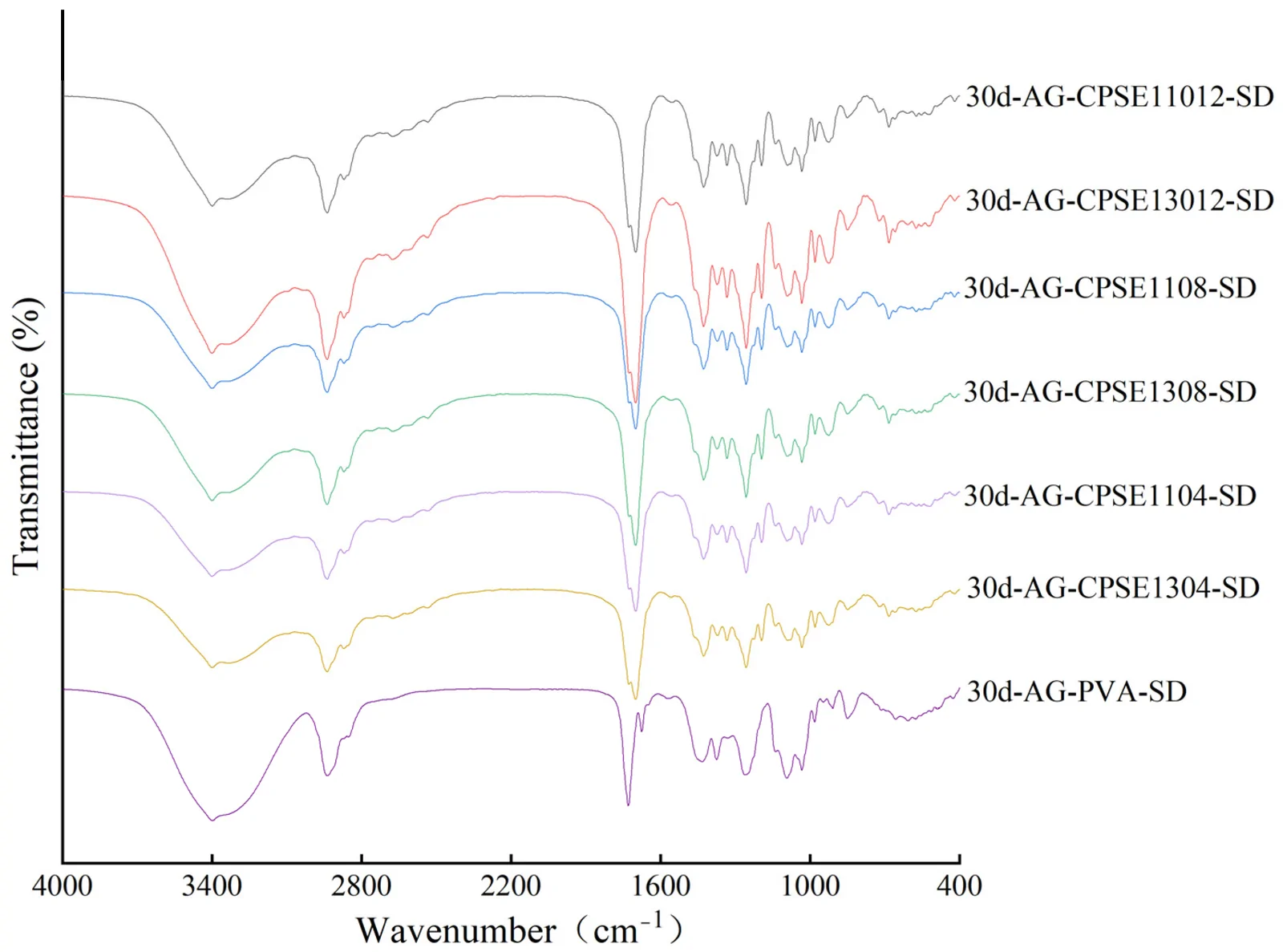
FTIR spectra of AG-SDs after 30 days of storage at 40 ± 2 °C/75 ± 5% RH.

Figure 8 shows the FTIR spectra of AG-SDs after 30 days of storage at 40 ± 2 °C/75 ± 5% RH. Among them, the –OH stretching band (3200–3600 cm^−1^) is the most sensitive to changes in hydrogen-bonding interactions; therefore, its peak position (cm^−1^) was used as the main indicator for evaluating changes in intermolecular interactions before and after storage.

After 30 days of storage, the –OH bands of all AG-SDs shifted to lower wavenumbers, exhibiting a significant red shift (the specific red-shift wavenumbers are listed in Table 2). This was attributed to moisture absorption by the AG-SD system, where water molecules disrupted the interactions between AG and the carrier material. Compared with AG-PVA-SD, the degree of red shift in AG-CPSE-SD was significantly reduced. In particular, the changes in the –OH peak position of AG-CPSE130-SDs were all less than 5 cm^−1^ (4, 4, and 3 cm^−1^, respectively), suggesting that CPSE130, with its higher crosslinking degree and more compact structure, restricted molecular movement, thereby enhancing the stability of the intermolecular interactions between AG and CPSE130.

In addition, compared with the samples before storage, the C=O stretching band at 1700–1730 cm^−1^ and the C–O–C bands at 1000–1250 cm^−1^ of AG-SDs showed only slight changes after 30 days of storage, with no obvious shift or change in peak profile, further indicating that AG-CPSE-SD remained structurally relatively stable during storage, which is consistent with the conclusion drawn from the –OH band shifts.

#### 3.8.3. Recrystallization Stability

**Figure 9:**
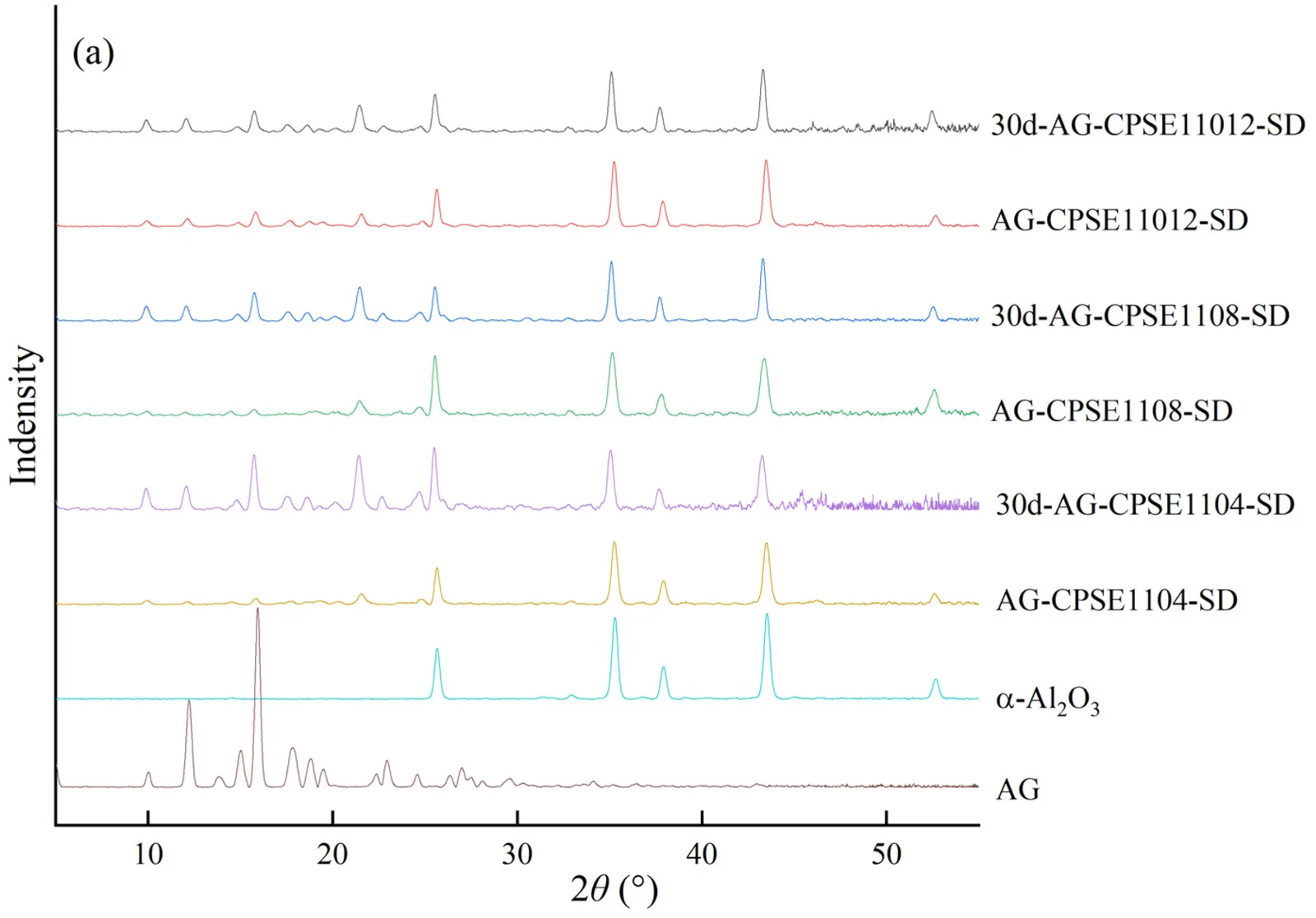

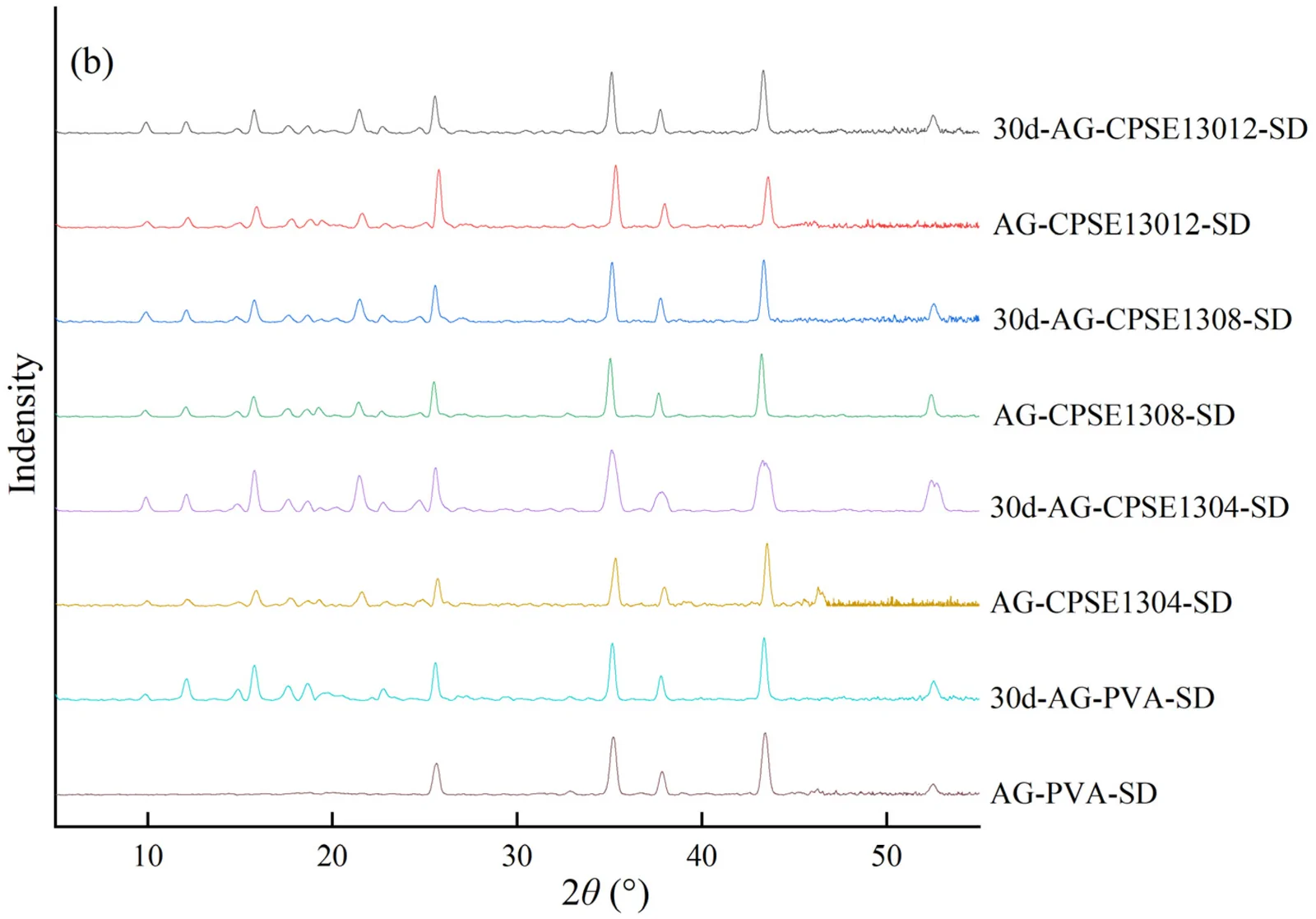
XRD diffractogram of AG-SDs before and after 30 days of storage at 40 ± 2 °C/75 ± 5% RH. (**a**) CPSE110 as carrier material; (**b**) CPSE130 as carrier material.

As shown in Figure 9, after 30 days of storage at 40 ± 2 °C/75 ± 5%RH, the AG characteristic diffraction peaks of both AG-CPSE-SDs and AG-PVA-SD were significantly enhanced. The *RC* of AG-SD was calculated using the internal standard method, and the results are presented in Table 3. As shown in Table 3, in the freshly prepared AG-SDs, the initial *RC* of AG-PVA-SD was 0. However, after 30 days, its increase in *RC* was the highest (Δ*RC* = 56.66%), which was significantly higher than that of AG-CPSE-SDs.

#### 3.8.4. Dissolution Stability

**Figure 10:**
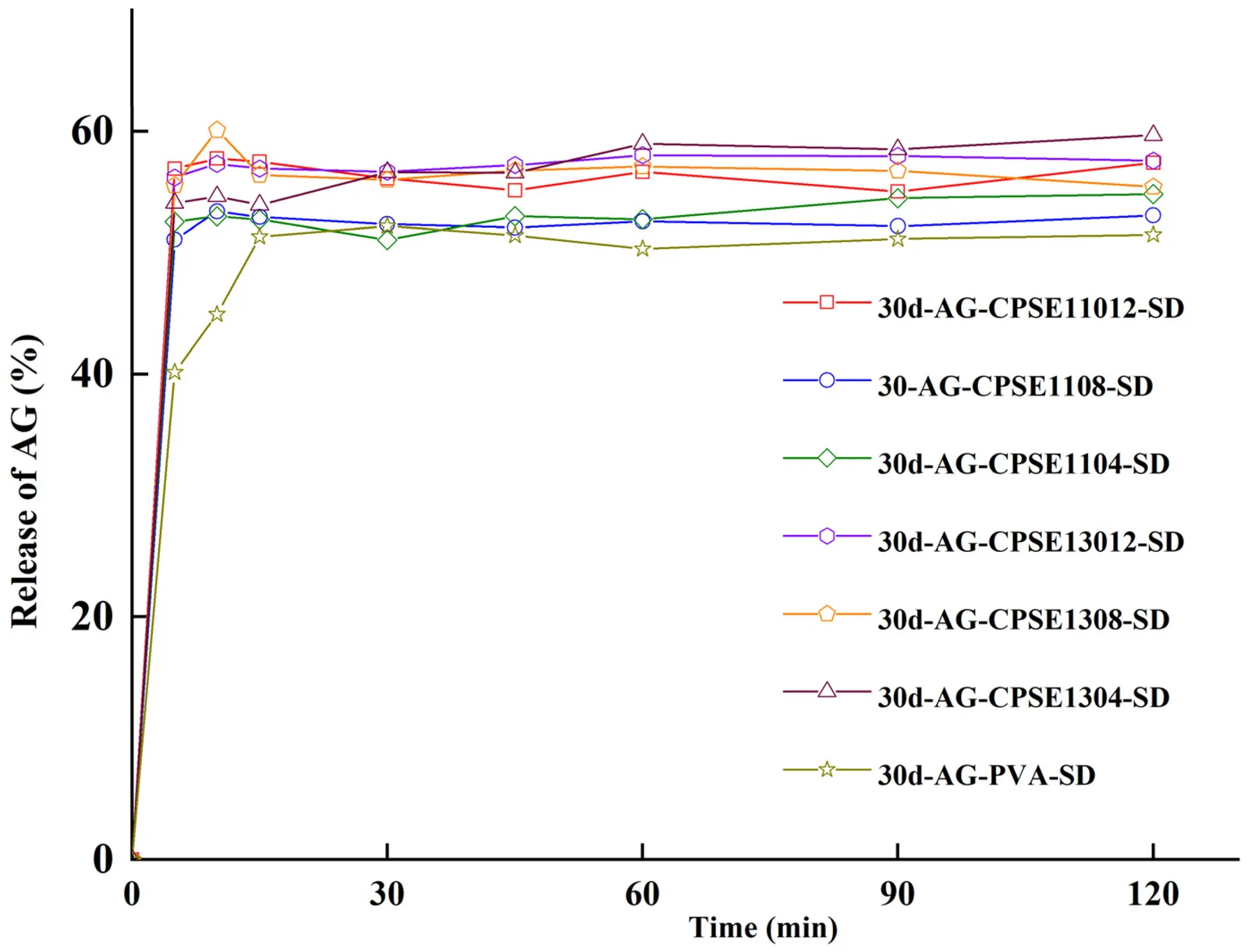
Dissolution profiles of AG-SDs before and after 30 days of storage at 40 ± 2 °C/75 ± 5% RH.

As shown in Figure 10 and Table 6, compared to the corresponding AG-SD, the dissolution curves of 30d-AG-PVA-SD, 30d-AG-CPSE1104-SD, and 30d-AG-CPSE1108-SD exhibited significant changes (ƒ_1_ > 15), which may be attributed to the larger increase in their *RC* and the higher lattice energy that must be overcome during dissolution.

## 4. Discussion

PVA is a polymer, while CPSE is a crosslinked polymer based on PVA. Compared with AG-PVA-SD prepared using PVA as the carrier material, AG-CPSE-SD prepared with the crosslinked polymer CPSE as the carrier material exhibited an increased *RC*, which was unfavorable for the dissolution of AG. However, both the surface free energy and its increase value were reduced in AG-CPSE-SD, and the hygroscopicity was also decreased, all of which were beneficial for improving the stability of the SD. Therefore, in this study, AG-SDs were stored under conditions of 40 ± 2 °C/75 ± 5%RH to further investigate the effect of the crosslinked polymer on the stability of AG-SD.

Hygroscopicity is one of the key factors affecting the stability of SDs [37,38]. After absorbing moisture, water acts as a plasticizer in SDs due to its extremely low *T*_g_, thereby reducing the *T*_g_ of SDs. Additionally, the unique structure of water molecules can disrupt the interactions between the drug and the polymer, significantly accelerating the recrystallization process of SDs.

Compared with AG-PVA-SD, the carrier material used in AG-CPSE-SD was a crosslinked polymer. Its network structure hindered the entry and diffusion of water molecules, thereby significantly improving the hygroscopic stability of AG-CPSE-SDs. Moreover, this network structure restricted the migration of drug molecules so that the recrystallization rate of the six AG-CPSE-SDs was significantly lower than that of AG-PVA-SD, in which water molecules disrupted the drug–carrier interactions (as shown in Table 2) and the carrier lacked a network structure to limit drug migration. A further comparison between AG-CPSE110-SDs and AG-CPSE130-SDs showed that although AG-CPSE130-SDs had a higher initial *RC*, the more compact structure of CPSE130 restricted the migration rate of AG molecules and thus effectively reduced the recrystallization rate. Consequently, after 30 days of storage at 40 ± 2 °C/75 ± 5%RH, the increase in *RC* of AG-CPSE130-SDs was less than 10%, indicating better recrystallization stability than AG-PVA-SD and AG-CPSE110-SDs.

**Figure 11:**
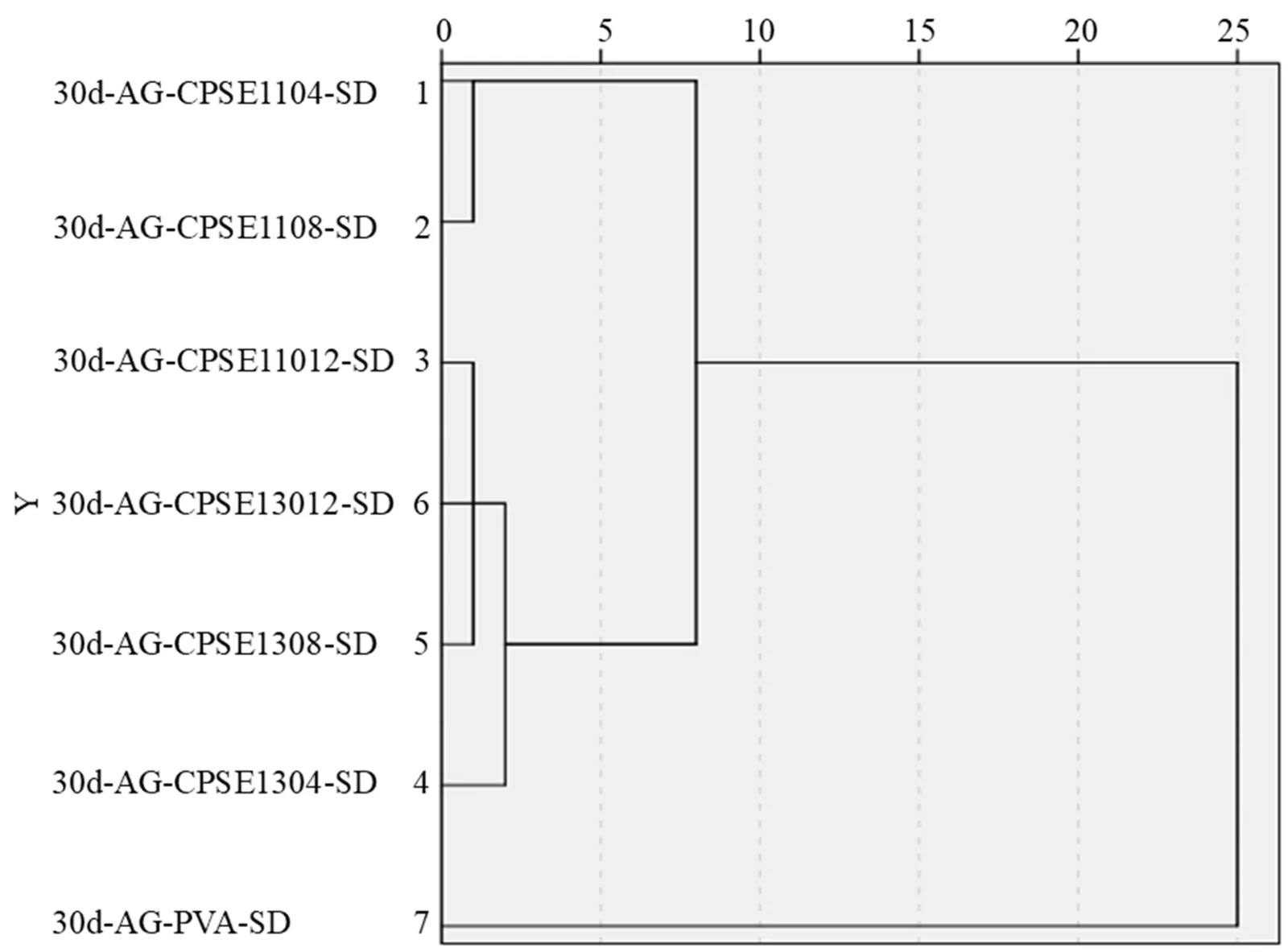
Cluster analysis dendrogram with AG dissolution percentage at different time as variable.

To further investigate the effect of crosslinking on the dissolution stability of AG-SDs, a cluster analysis was performed on the dissolution profiles of all 30d-AG-SDs. The AG dissolution percentage at different times was used as the clustering variable, and the samples were clustered using the between-groups linkage method and squared Euclidean distance. Figure 11 presents the dendrogram of the cluster analysis results. As shown in Figure 11, when the distance was less than 8, the samples could be divided into three groups: 30d-AG-PVA-SD in the first group, 30d-AG-CPSE1104-SD and 30d-AG-CPSE1108-SD in the second group, and the remaining samples in the third group. The cluster analysis results indicated that the first group (AG-SD with a non-crosslinked polymer as the carrier material) and the second group (AG-SD with CPSE1104 and CPSE1108 as carrier materials with lower crosslinking degree) could not maintain the dissolution stability of the samples, which was consistent with the calculation results of ƒ_1_. In contrast, CPSE11012, CPSE1304, CPSE1308, and CPSE13012, with a higher crosslinking degree and their compact network structures, could effectively hinder the migration and recrystallization of AG molecules within the SDs, thereby exhibiting better AG dissolution stability. When the distance was less than 2, 30d-AG-CPSE1304-SD was independently separated from the third group, indicating that compared to 30d-AG-CPSE11012-SD, 30d-AG-CPSE1308-SD and 30d-AG-CPSE13012-SD, 30d-AG-CPSE1304-SD had a higher dissolution percentage.

## 5. Conclusions

The results show that at day 0, AG-CPSE-SDs exhibited a higher RC, a lower surface free energy and a smaller increment in surface free energy, and reduced hygroscopicity compared with AG-PVA-SD. The dissolution curves of AG-CPSE1104-SD, AG-CPSE1108-SD and AG-CPSE1304-SD were equivalent to that of AG-PVA-SD, whereas those of AG-CPSE11012-SD, AG-CPSE1308-SD and AG-CPSE13012-SD were not. This indicates that at a high crosslinking degree, the crosslinked structure slows water penetration, reduces the solubility of the system and hinders drug dissolution in the crosslinked polymer-SD system. After 30 days of storage under high temperature and high humidity, AG-CPSE1304-SD, AG-CPSE1308-SD and AG-CPSE13012-SD exhibited good hygroscopic stability, intermolecular interaction stability, recrystallization stability and dissolution stability, owing to the compact network structure of the crosslinked polymer CPSE130, which restricted water entry and diffusion and slowed down the migration and recrystallization rates of AG.

Among the AG-CPSE-SDs using crosslinked polymers as carrier materials, AG-CPSE1304-SD simultaneously ensured effective dissolution of the poorly soluble drug AG and exhibited good stability. This study demonstrates that by controlling the crosslinking conditions and adjusting the crosslinking degree of crosslinked polymers, it is possible to improve the stability of SDs while ensuring the solubility of the system and the effective dissolution of the poorly soluble drug.

## Figures and Tables

**Table 1:** *γ*_l_, $\gamma_{l}^{p}$, $\gamma_{l}^{d}$ of H_2_O and CH_2_I_2_ (mJ/m^2^).

	*γ*_l_	$\gamma_{l}^{p}$	$\gamma_{l}^{d}$
H_2_O	72.8	51.0	21.8
CH_2_I_2_	50.8	0	50.8

**Table 2:** OH peak position of AG in AG-PM and AG-SD.

Sample	-OH Peak Position (cm^−1^)	Sample	-OH Peak Position (cm^−1^)		
			0 Day	30 Days	∆
AG-CPSE1104-PM	3344	AG-CPSE1104-SD	3358	3347	−11
AG-CPSE1108-PM	3332	AG-CPSE1108-SD	3348	3341	−7
AG-CPSE11012-PM	3324	AG-CPSE11012-SD	3346	3337	−9
AG-CPSE1304-PM	3335	AG-CPSE1304-SD	3344	3339	−5
AG-CPSE1308-PM	3322	AG-CPSE1308-SD	3334	3332	−2
AG-CPSE13012-PM	3315	AG-CPSE13012-SD	3329	3326	−3
AG-PVA-PM	3316	AG-PVA-SD	3370	3348	−22
AG	3315				

**Table 3:** *RC* (%) of AG-SDs.

Sample	*RC* (%)		
	0 d	30 d	∆
AG-CPSE1104-SD	10.75	57.65	46.90
AG-CPSE1108-SD	12.62	45.80	33.18
AG-CPSE11012-SD	19.83	41.12	21.29
AG-CPSE1304-SD	20.96	30.77	9.87
AG-CPSE1308-SD	25.58	32.86	8.59
AG-CPSE13012-SD	27.34	30.12	2.30
AG-PVA-SD	0	56.66	56.66

**Table 4:** Contact angle, $\gamma_{s}$ (mJ/m^2^), $\gamma_{s}^{p}$ (mJ/m^2^) and $\gamma_{s}^{d}$ (mJ/m^2^) of AG-SDs and AG-PMs.

Sample	Water Contact Angle (°)	$\gamma_{s}^{p}$	$\gamma_{s}^{d}$	$\gamma_{s}$	$\Delta \gamma_{s}$
AG-CPSE1104-PM	72.82	6.89	37.00	43.89	
AG-CPSE11004-SD	68.75	8.33	38.53	46.86	2.97
AG-CPSE1108-PM	81.30	3.68	36.52	40.19	
AG-CPSE1108-SD	70.15	8.98	34.34	43.32	3.13
AG-CPSE11012-PM	82.08	3.51	36.05	39.56	
AG-CPSE11012-SD	76.73	5.13	37.49	42.61	3.05
AG-CPSE1304-PM	78.37	5.45	33.60	39.05	
AG-CPSE1304-SD	69.35	7.91	38.98	46.89	7.84
AG-CPSE1308-PM	85.10	2.45	36.82	39.27	
AG-CPSE1308-SD	73.15	6.64	37.40	44.04	4.77
AG-CPSE13012-PM	85.82	2.05	38.10	40.15	
AG-CPSE13012-SD	75.45	5.37	38.55	43.92	3.77
AG-PVA-PM	100.25	0.18	33.20	33.38	
AG-PVA-SD	63.98	10.76	38.42	49.18	15.80

**Table 5:** *f*_1_ of AG-CPSE-SD moisture absorption curves.

*R*_t_	*T*_t_	*f*_1_
AG-CPSE1104-SD	AG-CPSE1108-SD	11.88
AG-CPSE1108-SD	AG-CPSE11012-SD	24.44
AG-CPSE1304-SD	AG-CPSE1308-SD	12.38
AG-CPSE1308-SD	AG-CPSE13012-SD	34.25
AG-CPSE1104-SD	AG-CPSE1304-SD	9.53
AG-CPSE1108-SD	AG-CPSE1308-SD	12.65
AG-CPSE11012-SD	AG-CPSE13012-SD	17.49

**Table 6:** *f*_1_ of AG-SD dissolution curves.

*T*_t_	*R*_t_	*T*_t_	*R*_t_
	AG-PVA-SD		Corresponding AG-SD
AG-CPSE1104-SD	12.60	30d-AG-CPSE1104-SD	16.23
AG-CPSE1108-SD	14.67	30d-AG-CPSE1108-SD	15.49
AG-CPSE11012-SD	15.88	30d-AG-CPSE11012-SD	7.12
AG-CPSE1304-SD	14.58	30d-AG-CPSE1304-SD	8.43
AG-CPSE1308-SD	16.94	30d-AG-CPSE1308-SD	5.32
AG-CPSE13012-SD	16.93	30d-AG-CPSE13012-SD	4.49
AG-PVA-SD	-	30d-AG-PVA-SD	29.29

## Data Availability

The data presented in this study are available on request from the corresponding author.
